# Reducing selection bias in case-control studies from rare disease registries

**DOI:** 10.1186/1750-1172-6-61

**Published:** 2011-09-12

**Authors:** J Alexander Cole, John S Taylor, Thomas N Hangartner, Neal J Weinreb, Pramod K Mistry, Aneal Khan

**Affiliations:** 1 Biomedical Data Sciences and Informatics, Genzyme, a Sanofi Company, 500 Kendall Street, Cambridge, MA, 02142, USA; 2 Biomedical, Industrial & Human Factors Engineering, Wright State University, 3640 Col. Glenn Highway, 207 Russ Egr. Center, Dayton, OH, 45435, USA; 3 University Research Foundation for Lysosomal Storage Diseases, Northwest Oncology Hematology Associates PA, 8170 Royal Palm Boulevard, Coral Springs, FL, 33065, USA; 4 Pediatric Gastroenterology and Hepatology Yale University School of Medicine, PO Box 208064, 333 Cedar Street; LMP 4093, New Haven, CT, 06520, USA; 5 University of Calgary, Alberta Children's Hospital, 2888 Shaganappi Tr NW, 3rd Floor Metabolic Clinic, Alberta, Calgary, Canada

## Abstract

**Background:**

In clinical research of rare diseases, where small patient numbers and disease heterogeneity limit study design options, registries are a valuable resource for demographic and outcome information. However, in contrast to prospective, randomized clinical trials, the observational design of registries is prone to introduce selection bias and negatively impact the validity of data analyses.

The objective of the study was to demonstrate the utility of case-control matching and the risk-set method in order to control bias in data from a rare disease registry. Data from the International Collaborative Gaucher Group (ICGG) Gaucher Registry were used as an example.

**Methods:**

A case-control matching analysis using the risk-set method was conducted to identify two groups of patients with type 1 Gaucher disease in the ICGG Gaucher Registry: patients with avascular osteonecrosis (AVN) and those without AVN. The frequency distributions of gender, decade of birth, treatment status, and splenectomy status were presented for cases and controls before and after matching. Odds ratios (and 95% confidence intervals) were calculated for each variable before and after matching.

**Results:**

The application of case-control matching methodology results in cohorts of cases (i.e., patients with AVN) and controls (i.e., patients without AVN) who have comparable distributions for four common parameters used in subject selection: gender, year of birth (age), treatment status, and splenectomy status. Matching resulted in odds ratios of approximately 1.00, indicating no bias.

**Conclusions:**

We demonstrated bias in case-control selection in subjects from a prototype rare disease registry and used case-control matching to minimize this bias. Therefore, this approach appears useful to study cohorts of heterogeneous patients in rare disease registries.

## Background

Rare diseases, exemplified by Gaucher disease, are defined as having a prevalence of fewer than 200,000 patients [1]. A major impediment to the study of these diseases is the scarcity of patients in any one city or country. Nevertheless, the global burden of patients affected by rare diseases is substantial: at least 30 million patients are estimated to suffer from one of the 7,000 rare diseases currently identified [2]. On average, each rare disease is estimated to afflict 4,200 patients [2]. Our search of the word 'registry' on clinicaltrials.gov as of 4 May 2011 identified 913 results.

Rare disease patient registries provide relatively large representative cohorts for clinical study. As a rule individual rare diseases are highly heterogeneous in phenotypic expression, which hinders optimal natural history or outcomes studies using data from rare disease registries. An excellent example of a rare disease registry is the International Collaborative Gaucher Group (ICGG) Gaucher Registry, which has been collecting patient data for 20 years. In fact, the ICGG Gaucher Registry is the prototype by which several disease registries have been created (Table 1).

**Table 1:** Rare Disease Registries[29]

Lysosomal Storage Diseases with Registries	Examples of Rare Diseases with Registries
• Fabry disease	• Adrenocortical Tumors

• Gaucher disease	• Alport Syndrome

• Hunter syndrome	• Epidermolysis Bullosa

• Mucopolysacchiridosis (MPS) type 1	• Inflammatory Breast Cancer

• Pompe disease	• Juvenile Rheumatoid Arthritis

	• Neuroendocrine Tumors

	• Neurological Autoimmune Disease

	• Neutropenia

	• Primary Ciliary Dyskines

	• Primary & Secondary Immunodeficiency

	• Smith Magenis Syndrome

	• Thrombotic Thrombocytopenia Purpura

	• Unexplained Cardiac Arrest

	• Vascular Anomalies Associated with Coagulapathy

	• Wilms Tumor Suppressor Gene Mutation (WT1) Associated Diseases

Randomized double-blind, placebo controlled clinical trials represent the highest category of evidence base for determining efficacy of treatments. For rare hereditary diseases, such as Gaucher disease, there are significant impediments to the design and conduct of adequately powered clinical trials. For example, rarity of the disease compounded by genetic and phenotypic heterogeneity hinders the development of appropriate subject groups for study that are controlled for factors such as age, sex, disease severity, and genotype. Moreover, following the introduction of an effective therapy, few patients remain treatment-naive for evaluation of alternative therapies, which may differ in mechanism of action and have overlapping effects. An additional consideration when evaluating long-term treatment outcomes is the chronic nature of many rare diseases, which often extends beyond the reasonable time span of a traditional clinical trial. As an alternative model, the Framingham heart study provides an example of the design and conduct of an observational cohort study designed to collect longitudinal data with the goal of studying health outcomes [3].

An important feature of disease registries is the potential to provide real-world data from the community [2]. Therefore, data from registries could complement data obtained from clinical trials to develop optimal standards of care for rare diseases. Indeed, data from the ICGG Gaucher Registry have been effectively used to demonstrate treatment outcomes in multiple disease compartments which have been used to develop a standard of care and expected treatment outcomes for Gaucher disease [4-6]. These have formed the basis for developing therapeutic goals [7] and to define endpoints for subsequent clinical trials of new therapeutic agents [8-10]. Analytical approaches used in these studies from the ICGG Gaucher Registry have included multivariate mixed-effects analyses [11], propensity scoring and non-linear effects modeling [12], and Poisson regression modeling to determine relative risk [13].

A major confounder with registry data is selection bias, which is inherent in the observational design of the registry and the flexibility accorded to contributors to determine which patients to include and what data to submit [14]. An approach to overcome such selection bias is the use of case-control matching, in which cases are selected based on the presence of a specific disease outcome and matched to controls that are identified to not have that outcome. These cases and controls are matched according to values for a set of background characteristics. However, this type of analysis requires a population sufficiently large to identify cases of interest and randomly selected controls. With almost 6,000 enrolled subjects, the ICGG Gaucher Registry is the largest worldwide registry for an inborn error of metabolism, and it becomes feasible to attempt case-control matching.

In this paper, the cases of interest are patients with skeletal avascular osteonecrosis (AVN), a serious and irreversible complication of Gaucher disease that occurs sporadically and unpredictably in a subset of patients. The set of matched controls are patients with type 1 Gaucher disease who did not develop AVN. By applying the risk-set method approach, we demonstrate the utility of the case-control matching method to identify case and control patients who have comparable distributions for four common parameters used in subject selection: gender, year of birth (age), treatment status, and splenectomy status. We conclude that selection bias in case-control selection of subjects from rare disease registries occurs and that this can be overcome through case-control matching to minimize bias. Therefore, application of this technique permits the study of treatment outcomes or natural history within rare disease registries.

## Methods

### International Collaborative Gaucher Group (ICGG) Gaucher Registry

The ICGG Gaucher Registry was started to track the clinical, demographic, genetic, biochemical and therapeutic characteristics of patients with Gaucher disease throughout the world, irrespective of disease severity, treatment status, or treatment choice [15]. An independent international group of physician experts in Gaucher disease provides scientific direction and governance of the Registry, with logistical support from Genzyme, a Sanofi Company (Cambridge, Massachusetts). Since its inception in 1991, with Institutional Review Board/Ethics Committee approvals, over 700 physicians from more than 60 countries have voluntarily submitted de-identified data on over 5,800 patients to the Registry.

### Study population

We identified all patients in the ICGG Gaucher Registry as of 1 October 2010, with type 1 Gaucher disease and reported treatment status including date of initiation of imiglucerase (Cerezyme^®^, Genzyme Corporation) or alglucerase (Ceredase^®^, Genzyme Corporation) treatment. Until early 2010, alglucerase and imiglucerase were the only commercially approved enzyme treatments for Gaucher disease. Alglucerase and imiglucerase have been shown to be therapeutically equivalent in a randomized, two-arm clinical trial [16]. For simplicity, these two treatments will be denoted as imiglucerase in this publication.

### Case identification

Based on data from the ICGG Gaucher Registry skeletal case report forms, we identified all patients with affirmative reports of AVN. Cases of AVN were typically ascertained through radiographic or magnetic resonance image (MRI) results. An affirmative report was based on the treating physician's review of the corresponding radiographic or MRI result. Each patient's earliest date of an affirmative report of AVN was considered to be the index date.

### Case-control matching

In order to quantify the association between risk factors with the onset of AVN, we initially sought to identify all patients without AVN as controls in our analysis. Following a review of characteristics between cases and controls, apparent differences between the groups according to gender, decade of birth, imiglucerase/alglucerase treatment status, and history of splenectomy were noted. Prior to the advent of imiglucerase, patients underwent splenectomy for relief of cytopenia and/or pressure symptoms; however, splenectomy itself has the potential to alter the phenotype and natural course of the disease [17,18]. Since these variables (gender, decade of birth, treatment status, history of splenectomy) may impact both the risk of AVN and also may be associated with other risk factors for AVN, we implemented a case-control matching algorithm using the risk-set method [19]. For each case of AVN, we identified all controls who matched on gender and year of birth (± five years). Among these matched controls, we then assigned their index date to be the same date as the AVN onset date for the corresponding case and excluded controls who were not followed-up in the ICGG Gaucher Registry as of that index date. We further determined whether the case and controls as of their index date had 1) initiated treatment with imiglucerase/alglucerase and 2) underwent prior splenectomy. For each individual case, we randomly selected up to five controls who matched on all four characteristics [20].

### Statistical analysis

We presented the frequency distributions of gender, decade of birth, treatment status, and splenectomy status for cases and controls before and after matching. We calculated odds ratios (and 95% confidence intervals) for each variable before and after matching and present the percent bias for each variable [21,22] using the formula below:

[(ARR-RR∕RR)]×100

where

ARR = Apparent exposure relative risk (i.e., before matching)

RR = 'True' or fully adjusted exposure relative risk (i.e., after matching)

An odds ratio of 1·00 indicates no difference in the distributions between cases and controls [23]. All analyses were conducted in SAS 9·1 (SAS Institute Inc., Cary, North Carolina, USA) in accordance with STrengthening the Reporting of OBservational studies in Epidemiology **(**STROBE) guidelines [24].

## Results

As of 1 October 2010, the ICGG Gaucher Registry contained a total of 5,894 patients. Of these, 5,156 patients met the study inclusion criteria: type 1 Gaucher disease, known treatment status, and known date of initiation of treatment. From this group of patients (n = 5,156), 176 patients had a history of AVN with no accompanying assessment or diagnosis dates reported to the Registry and were therefore excluded from the study. Of the remaining 4,980 patients, we identified 853 patients with reports of AVN and 4,127 patients without AVN.

Patient characteristics before matching are shown in Table 2. Before matching, the ratio of females to males was similar in both groups, with a slightly higher percentage of females in the control group. In contrast, before matching, a higher percentage of patients born in earlier decades (i.e. older patients with more years at risk) reported AVN compared to the group without AVN. Additionally, distributions of splenectomy and treatment status were substantially different between case and control patients, as indicated by odds ratios of 3·21 for splenectomy status and 6·09 for treatment status.

**Table 2:** Patient Characteristics Before Matching

	Patients with Avascular Necrosis	Patients without Avascular Necrosis	Odds Ratio	95% Confidence Interval
**Before Matching**	853	4127		

**Gender, n (%)**				

Male	423 (49·6)	1899 (46·0)	Reference*	

Female	430 (50·4)	2228 (54·0)	0·89	(0·78, 1·02)

**Year of Birth, n (%)**				

1910 - < 1920	4 (0·5)	34 (0·8)	0·49	(0·18, 1·31)

1920 - < 1930	21 (2·5)	125 (3·0)	0·66	(0·42, 1·05)

1930 - < 1940	75 (8·8)	198 (4·8)	1·27	(0·96, 1·68)

1940 - < 1950	114 (13·4)	384 (9·3)	1·06	(0·83, 1·35)

1950 - < 1960	146 (17·1)	529 (12·8)	Reference*	

1960 - < 1970	147 (17·2)	590 (14·3)	0·92	(0·73, 1·16)

1970 - < 1980	156 (18·3)	665 (16·1)	0·88	(0·70, 1·10)

1980 - < 1990	129 (15·1)	659 (16·0)	0·76	(0·60, 0·96)

1990 - < 2000	58 (6·8)	633 (15·3)	0·39	(0·29, 0·53)

2000 - < 2010	3 (0·4)	310 (7·5)	0·04	(0·01, 0·14)

**Treatment Status, n (%)**				

Untreated	35 (4·1)	995 (24·1)	Reference*	

Treated	818 (95·9)	3132 (75·9)	6·09	(4·34, 8·55)

**Splenectomy Status, n (%)**				

Spleen Intact	438 (51·3)	3407 (82·6)	Reference*	

Splenectomized	415 (48·7)	720 (17·4)	3·21	(2·81, 3·67)

* "Reference" indicates group used for odds ratios comparison.

**Figure 1:**
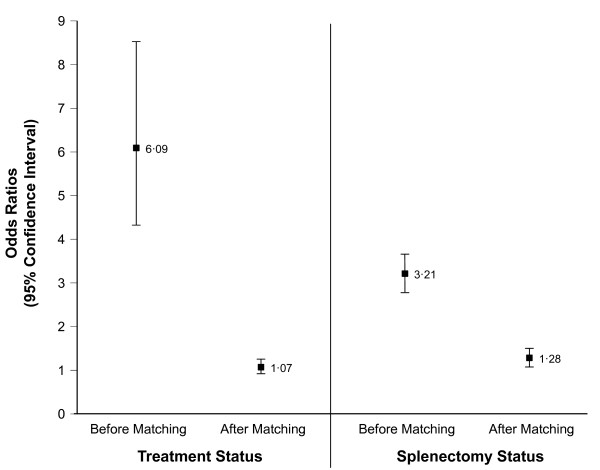
**Odds Ratios in Subjects With and Without Avascular Osteonecrosis Before and After Matching**.

In general, matching resulted in odds ratios of approximately 1·00 as seen in Table 3. After matching, the distributions of patients born in each decade in both groups were more comparable. For splenectomy status and treatment status, where differences in distributions before matching were apparent, the percent bias was ((3·21 - 1·32)/1·32) × 100 = 143·2% and ((6·09 - 1·10)/1·10) × 100 = 453·6%, respectively (Figure 1).

**Table 3:** Patient Characteristics After Matching

	Patients with Avascular Necrosis	Patients without Avascular Necrosis	Odds Ratio	95% Confidence Interval
**After Matching**	672	2390		

**Gender, n (%)**				

Male	323 (48·1)	1139 (47·7)	Reference*	

Female	349 (51·9)	1251 (52·3)	0·99	(0·85,1·15)

**Year of Birth, n (%)**				

1910 - < 1920	3 (0·4)	17 (0·7)	0·66	(0·21,2·07)

1920 - < 1930	17 (2·5)	78 (3·3)	0·79	(0·47,1·31)

1930 - < 1940	64 (9·5)	163 (6·8)	1·24	(0·91,1·68)

1940 - < 1950	88 (13·1)	299 (12·5)	1·00	(0·76,1·32)

1950 - < 1960	114 (17·0)	387 (16·2)	Reference*	

1960 - < 1970	89 (13·2)	379 (15·9)	0·84	(0·63,1·10)

1970 - < 1980	124 (18·5)	404 (16·9)	1·03	(0·80,1·33)

1980 - < 1990	115 (17·1)	354 (14·8)	1·08	(0·83,1·40)

1990 - < 2000	55 (8·2)	276 (11·5)	0·73	(0·53,1·01)

2000 - < 2010	3 (0·4)	33 (1·4)	0·37	(0·12,1·15)

**Treatment Status, n (%)**				

Untreated	322 (47·9)	1197 (50·1)	Reference*	

Treated	350 (52·1)	1193 (49·9)	1·07	(0·92,1·24)

**Splenectomy Status, n (%)**				

Spleen intact	447 (66·5)	1752 (73·3)	Reference*	

Splenectomized	225 (33·5)	638 (26·7)	1·28	(1·09,1·51)

* "Reference" indicates group used for odds ratios comparison.

## Discussion

Registries for the study of rare diseases serve to create pooled patient populations that are sufficiently large for robust statistical analysis. However, studies based on registry databases are vulnerable to bias. For example, domains captured in the database may differ from center to center; patients with less severe disease may not be enrolled or, if enrolled, may have fewer data collected. In addition, the data may be incomplete. Verification of the quality or completeness of the data may be lacking and there is no systematic evaluation of statistical methods to generate an unbiased dataset from registry data. Nevertheless, as many long-term studies [25-27] have demonstrated in a variety of diseases, having longitudinal data is critical to understanding the natural history or response to treatment of a chronic disease. This type of data is often analyzed using case-control methodology.

However, case-control studies in patients with rare diseases, whether performed in individual large clinics or through disease registries, are inherently vulnerable to bias. Chronic diseases, such as Gaucher disease, are highly heterogeneous, and the phenotype can vary depending on the age of onset, age of the patient, adjunct therapies, genotype, access to health-care resources, and environmental factors. Patients with milder disease tend to have less contact with specialty clinics and less frequent and intensive follow-up; many are not diagnosed for several years [28]. When more than one control is identified that matches to each case, there has been no validation to our knowledge, whether non-random selection of a control, pooling all controls, or selecting a group of controls are valid methods to reduce selection bias. Therefore, selecting an unbiased control group is not simply a matter of finding subjects who are negative for the disease variable being studied, and arbitrary selection of controls or pooling of controls does not obviate having a biased control group that may lead to an erroneous conclusion. The method we used permitted appropriate risk-set selection and subsequent matching, and it circumvented the challenge of clinical heterogeneity in observational registries. However, it is applicable only in the context of a large, well annotated patient cohort combined with extensive follow-up data.

This study shows that some biases can be successfully minimized in an observational database such as the ICCG Registry by using case-control matching and a modified risk-set method approach. Applying this established method to registry data, we demonstrated the effective use of the case-control matching method to yield cohorts of case and control patients who have comparable distributions for four common areas used in subject selection: gender, year of birth (age), treatment status, and splenectomy status. The results after matching showed odds ratios close to one, which indicates no difference or bias between cases and controls on these matching variables. Skeletal avascular osteonecrosis was selected for this analysis because it is a complication of type 1 Gaucher disease associated with serious acute and chronic morbidity[13], but it is a difficult target to study because it occurs sporadically and unpredictably. The matched patients now constitute a resource for further analysis. In this cohort, other risk factors can now be studied without introducing bias due to differences in age, gender, treatment status, and splenectomy status.

In this study, the main outcome variable was the change in odds ratios. The odds ratios indicate the amount of bias in the groups. The largest changes were observed for treatment status and splenectomy status. This difference may be due to several factors. One factor is that many of the controls, even though they were not symptomatic for the variable in question, were receiving imiglucerase therapy. Because biased selection of controls may over or under represent the variables in case-control pairs, having more controls than cases may have made it appear as if AVN was more likely to occur in younger patients or subjects without a history of imiglucerase therapy or who underwent a prior splenectomy. Having randomly matched controls, the cases and controls were numerically equally represented, thus reducing the bias. The purpose of having matched data is to reduce the finding of any such relationship due to biased case or control selection.

The practical application of this technique is to validate that case-control studies have a minimized bias in subject selection, which provides researchers with an analytical tool to test their hypotheses of interest. This study has demonstrated the use of case-control matching to reduce the bias between groups. We conclude that bias in case-control selection in subjects from rare disease registries can occur, and case-control matching is one method to minimize this bias.

## Conclusions

This study shows that some biases can be successfully minimized in an observational database such as the ICCG Gaucher Registry by using case-control matching and a modified risk-set method approach.

## Competing interests

Aneal Khan, Pramod Mistry and Neal Weinreb receive honoraria and expense reimbursement for serving on the Board of Advisors of the ICGG Gaucher Registry; travel reimbursements and/or honoraria and/or research support from Genzyme, a Sanofi Company, Shire Pharmaceuticals, Amicus Therapeutics, and Actelion. Aneal Khan and Neal Weinreb do not hold any financial interest in any pharmaceutical company. Thomas Hangartner receives travel reimbursement and/or honoraria for speaking engagements from Genzyme, a Sanofi Company, and Shire Pharmaceuticals. John Taylor and J. Alexander Cole are employees of Genzyme, a Sanofi Company.

Aneal Khan, Pramod Mistry, Neal Weinreb and Thomas Hangartner did not receive funding for this study.

## Authors' contributions

AK was responsible for the hypothesis, overall concept, analyses, and data interpretation. He wrote the first draft, edited, and oversaw the writing of the manuscript. The research hypothesis was developed as an independent research question prior to joining the ICGG. AK presented a research request to the ICGG Gaucher Registry in order to test his hypothesis.

TH assisted in hypothesis development, data interpretation, and editing the manuscript.

JAC was primarily responsible for the overall epidemiologic design and statistical analyses, including the overall concept, data interpretation, and drafting and editing the manuscript.

JST was primarily responsible for the overall statistical analyses, including the data interpretation, and drafting and editing the manuscript.

PKM assisted in hypothesis development, editing the manuscript, and interpretation of data.

NJW assisted in hypothesis development, writing and editing the manuscript, and interpretation of data.

All authors read and approved the final manuscript.

## Authors' information

AK is an Assistant Professor of Medical Genetics and Pediatrics at the University of Calgary at Alberta Children's Hospital. His primary work is in the clinical management of patients with inborn errors of metabolism, including Gaucher disease, in addition to clinical research in the same area.

TNH is a Distinguished Professor of Biomedical Engineering, Medicine & Physics at Wright State University in Dayton, OH. His long-term interests in non-invasive, quantitative assessment of bone resulted in the invitation to participate in the data analysis and subsequent drafting of this manuscript.

JAC is Director, Epidemiology at Genzyme, a Sanofi Company, where he participates in the design and conduct of data analysis from disease registries, including the ICGG Gaucher Registry. He holds a Doctor of Science degree in Epidemiology.

JST is a Senior Biostatistician at Genzyme, a Sanofi Company, where he participates in the design and conduct of data analysis from the ICGG Gaucher Registry. He holds a Master of Arts degree in Statistics.

PKM is Professor and Chief, National Gaucher Disease Treatment Center at Yale School of Medicine. He has major clinical and research interests in Gaucher disease. He is a member of the Scientific Board of ICGG Gaucher Registry and his participation in the study derives from this role.

NJW is Voluntary Associate Professor of Medicine at the Miller School of Medicine of the University of Miami and Director of the University Research Foundation for Lysosomal Storage Diseases (unaffilliated with the University of Miami). He has had a research and clinical interest in Gaucher disease for 44 years. NJW is the chair of the North American Scientific Board of ICGG Gaucher Registry and co-chair of the International ICGG Board. His participation in the study derives from these roles.
